# The Antidepressant 5-HT_2A_ Receptor Antagonists Pizotifen and Cyproheptadine Inhibit Serotonin-Enhanced Platelet Function

**DOI:** 10.1371/journal.pone.0087026

**Published:** 2014-01-23

**Authors:** Olivia A. Lin, Zubair A. Karim, Hari Priya Vemana, Enma V. P. Espinosa, Fadi T. Khasawneh

**Affiliations:** Department of Pharmaceutical Sciences, College of Pharmacy, Western University of Health Sciences, Pomona, California, United States of America; University of Kentucky, United States of America

## Abstract

There is considerable interest in defining new agents or targets for antithrombotic purposes. The 5-HT_2A_ receptor is a G-protein coupled receptor (GPCR) expressed on many cell types, and a known therapeutic target for many disease states. This serotonin receptor is also known to regulate platelet function. Thus, in our FDA-approved drug repurposing efforts, we investigated the antiplatelet activity of cyproheptadine and pizotifen, two antidepressant 5-HT_2A_ Receptor antagonists. Our results revealed that cyproheptadine and pizotifen reversed serotonin-enhanced ADP-induced platelet aggregation *in vitro* and *ex vivo*. And the inhibitory effects of these two agents were found to be similar to that of EMD 281014, a 5-HT_2A_ Receptor antagonist under development. In separate experiments, our studies revealed that these 5-HT_2A_ receptor antagonists have the capacity to reduce serotonin-enhanced ADP-induced elevation in intracellular calcium levels and tyrosine phosphorylation. Using flow cytometry, we also observed that cyproheptadine, pizotifen, and EMD 281014 inhibited serotonin-enhanced ADP-induced phosphatidylserine (PS) exposure, P-selectin expression, and glycoprotein IIb-IIIa activation. Furthermore, using a carotid artery thrombosis model, these agents prolonged the time for thrombotic occlusion in mice *in vivo*. Finally, the tail-bleeding time was investigated to assess the effect of cyproheptadine and pizotifen on hemostasis. Our findings indicated prolonged bleeding time in both cyproheptadine- and pizotifen-treated mice. Notably, the increases in occlusion and bleeding times associated with these two agents were comparable to that of EMD 281014, and to clopidogrel, a commonly used antiplatelet drug, again, in a fashion comparable to clopidogrel and EMD 281014. Collectively, our data indicate that the antidepressant 5-HT_2A_ antagonists, cyproheptadine and pizotifen do exert antiplatelet and thromboprotective effects, but similar to clopidogrel and EMD 281014, their use may interfere with normal hemostasis.

## Introduction

Platelets are specialized anucleated cells that directly contribute to, and regulate hemostasis. Hemostasis is a physiological process that stops bleeding upon blood vessel injury. The inappropriate activation of hemostatic mechanisms and unrestrained platelet aggregation may lead to the development of thromboembolic events [1]–[4]. Therefore, it is critical to understand the mechanisms of platelet activation, in order to define novel pharmacological agents to reduce the adverse outcomes of unrestrained platelet activities.

Serotonin, or 5-hydroxytryptamine (5-HT), plays a key role in the development of arterial thrombosis [5]. It is predominantly synthesized/secreted into the blood stream by enterochromaffin cells in the gastrointestinal tract, and is rapidly taken up and stored in platelet dense granules [6]. As long as platelets do not aggregate, peripheral blood contains little or no free serotonin [7]. Upon platelet activation at the site of vessel injury, 5-HT is released from the dense granules in platelets. Serotonin or 5-HT by itself is a weak activator of platelet aggregation but it amplifies aggregation induced by other agonists including collagen, ADP, and epinephrine [8], [9]. Previous studies have shown a significant serotonin-dependent increase in platelet aggregation in depressed patients compared with controls, indicating serotonin plays an important role in the genesis of occlusive diseases [10], [11].

G protein coupled receptors (GPCRs) in platelets have been extensively studied to identify targets for treating a multitude of cardiovascular events. One such receptor is the serotonin 5-HT_2A_ receptor, which belongs to the 5-HT_2_ receptor family. The 5-HT_2A_ receptors are of significant clinical interest because they are involved in the mediation of mental disorders [12], [13], and many cardiovascular diseases [14], [15]. To this end, it has been previously shown that inhibition of serotonin 5-HT_2A_ receptor can improve coronary patency in *in-vivo* model of recurrent thrombosis [16]. Previous studies have also reported platelet aggregation is enhanced (e.g., hyperactive 5-HT_2A_ receptor signaling) in depressed patients [17]–[21]. This is because traditional therapies focus on elevating serotonin levels, but this approach can have serious side effects, including increased risk of serotonin syndrome and cardiovascular-related adverse events [22], [23].

Despite the crucial role of serotonin and 5-HT_2A_ receptor activation in platelet function, there are currently no 5-HT_2A_ receptor antagonists approved by the Food and Drug Administration (FDA), for treatment of arterial thrombosis [24]. This is an important issue given the limitations of current antiplatelet therapies. Based on these considerations, we sought to investigate whether conventional FDA-approved antidepressant drugs, namely cyproheptadine and pizotifen, can be repurposed to ameliorate serotonin receptor-dependent platelet aggregation and thrombogenesis [25]–[27]. Our studies revealed that these drugs do have the capacity to inhibit serotonin-enhanced ADP-induced platelet aggregation *in vitro*, and *ex vivo*; similar to EMD 281014, another potent and selective 5-HT_2A_ receptor antagonist [28]–[30]. These drugs also have the capacity to inhibit serotonin-enhanced ADP-induced elevation in intracellular calcium and tyrosine phosphorylation. We also observed that serotonin-enhanced ADP-stimulated platelet phosphatidylserine (PS) exposure, P-selectin expression, and glycoprotein (GP) IIb-IIIa activation were inhibited by cyproheptadine, pizotifen and EMD 281014, *in vitro*. Moreover, cyproheptadine and pizotifen were found to significantly prolong occlusion time in mouse thrombosis model, but normal hemostasis may also be interfered, as demonstrated in tail bleeding time experiments. Notably, the *in vivo* activities of cyproheptadine and pizotifen were determined to be comparable to that of the clinically-relevant and commonly prescribed antithrombotic drug, clopidogrel.

## Results

### Cyproheptadine and Pizotifen Inhibit Serotonin-enhanced ADP-induced Human Platelet Aggregation *in vitro*

**Figure 1:**
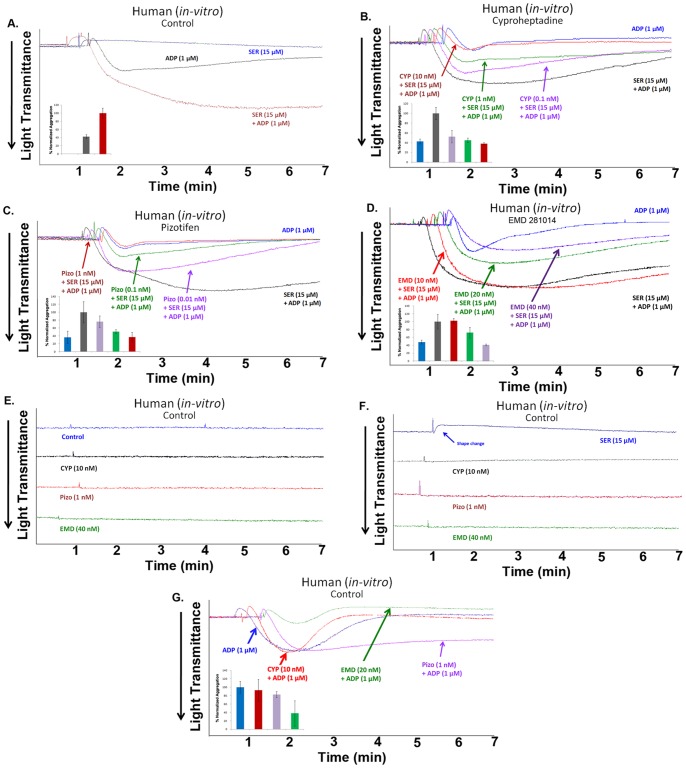
Cyproheptadine and pizotifen inhibit serotonin-enhanced ADP-induced human platelet aggregation *in vitro.* (A) Human PRP was stimulated with submaximal concentration of ADP (1 µM) in the presence or absence of serotonin (15 µM). (B) Human PRP was pre-incubated with increasing doses of cyproheptadine (0.1–10 nM) for 1 min and activated with ADP (1 µM) and serotonin (15 µM). (C) Human PRP was pre-incubated with increasing doses of pizotifen (0.01–1 nM) for 1 min and activated with ADP and serotonin. (D) Human PRP was pre-incubated with increasing doses of EMD 281014 (10–40 nM) for 1 min and activated with ADP and serotonin. (E) Human PRP was treated with the highest concentrations of cyproheptadine (10 nM), pizotifen (1 nM), and EMD 281014 (40 nM) used in previous experiments, in the absence of agonists. (F) Human PRP was pre-incubated for 1 min with cyproheptadine (10 nM), pizotifen (1 nM), and EMD 281014 (40 nM) for 1 min and activated with serotonin (15 µM). (G) Human PRP was pre-incubated with cyproheptadine (10 nM), pizotifen (1 nM), and EMD 281014 (40 nM) for 1 min and activated with ADP (1 µM). Inset shows quantification of the data. Each experiment was repeated at least 3 times, with blood obtained from three separate donors.

As a known weak activator of platelet aggregation [8], [31], serotonin did not induce platelet aggregation in human PRP (**Fig. 1A**). On the other hand, weak and reversible platelet aggregation was observed when platelets were stimulated with submaximal concentration of ADP (i.e., 1 µM). Simultaneous addition of serotonin (15 µM) resulted in significant potentiation of platelet aggregation induced by 1 µM of ADP (**Fig. 1A**), demonstrating that serotonin has the ability to enhance ADP-induced platelet aggregation. Next, we investigated whether the antidepressant 5-HT_2A_ receptor antagonists, namely cyproheptadine and pizotifen, can be repurposed as antiplatelet agents, and used to inhibit serotonin-enhanced ADP-induced platelet aggregation *in vitro.* Aggregation studies indicated that cyproheptadine (0.1–10 nM) and pizotifen (0.01–1 nM) have the capacity to dose-dependently inhibit serotonin-enhanced ADP-induced platelet aggregation (**Fig. 1B–1C**). The first set of control experiments was performed using EMD 281014, a potent and selective 5-HT_2A_ receptor antagonist; its antiplatelet activity has yet to be determined. Our result indicated that EMD 281014 (10–40 nM) also dose-dependently inhibited human platelet aggregation *in vitro* (**Fig. 1D**). To verify that cyproheptadine and pizotifen specifically antagonize serotonin-enhanced platelet function, and that they do not affect platelet activity in the absence of serotonin, a second series of experiments was performed. As expected, cyproheptadine (10 nM) pizotifen (1 nM), and EMD 281014 (40 nM) were found to inhibit (15 µM) serotonin-induced limited platelet activation (i.e., shape change; **Fig. 1E**), but neither agent (with the exception of EMD 281014) exerted any effects on ADP-induced platelet aggregation (**Fig. 1F**), or on non-stimulated resting platelets (**Fig. 1G**).

### Cyproheptadine and Pizotifen Inhibit Serotonin-enhanced U46619-induced Human Platelet Aggregation *in vitro*

**Figure 2:**
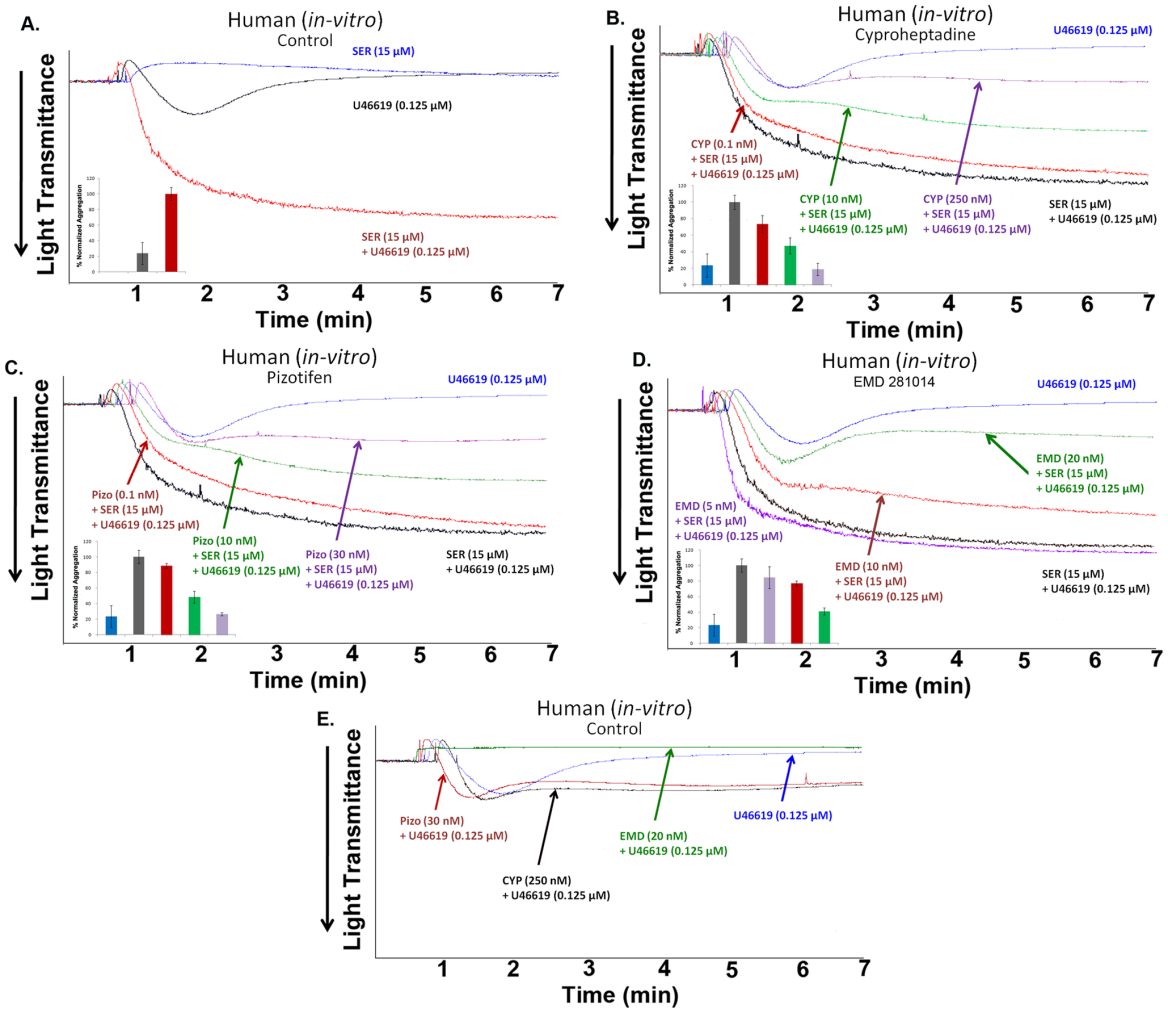
Cyproheptadine and pizotifen inhibit serotonin-enhanced U46619-induced human platelet aggregation *in vitro*. (A) Human PRP was stimulated with submaximal concentration of U46619 (0.125 µM) in the presence or absence of serotonin (15 µM). (B) Human PRP was pre-incubated with increasing doses of cyproheptadine (0.1–250 nM) for 1 min and activated with U46619 (0.125 µM) and serotonin (15 µM). (C) Human PRP was pre-incubated with increasing doses of pizotifen (0.1–30 nM) for 1 min and activated with 0.125 µM U46619 and 15 µM serotonin. (D) Human PRP was pre-incubated with increasing doses of EMD 281014 (5–20 nM) for 1 min and activated with 0.125 µM U46619 and 15 µM serotonin. (E) Human PRP was pre-incubated for 1 min with the highest concentrations of cyproheptadine (250 nM), pizotifen (30 nM), and EMD 281014 (20 nM) used in this set of experiments, and activated with U46619 (0.125 µM). Inset shows quantification of the data. Each experiment was repeated at least 3 times, with blood obtained from three separate donors.

Given that serotonin is a weak agonist that amplifies platelet aggregation responses, we investigated its effects on aggregation induced by another agonist, namely U46619 which is an agonist for the thromboxane receptor. Using U46619, a potent agonist of platelet aggregation, we were able to verify that serotonin has the ability to enhance platelet aggregation induced by submaximal concentration, i.e., 0.125 µM of U46619 (**Fig. 2A**). Again, separate aggregation studies revealed that cyproheptadine (0.1–250 nM) and pizotifen (0.1–30 nM) have the capacity to dose-dependently inhibit serotonin-enhanced U46619-induced platelet aggregation, *in vitro* (**Fig. 2B–2C**). EMD 281014 (5–20 nM) also has the capacity to dose-dependently inhibit serotonin-enhanced U46619-induced platelet aggregation (**Fig. 2D**). It was further demonstrated that each of the 5-HT_2A_ receptor antagonist used did not exert any effect on U46619-induced platelet aggregation, with the exception of EMD 281014 (**Fig. 2E**); this is consistent with what was observed with ADP (**Fig. 1E–1G**), and further supports that cyproheptadine and pizotifen do specifically inhibit serotonin-enhanced platelet function induced by multiple agonists.

**Figure 3:**
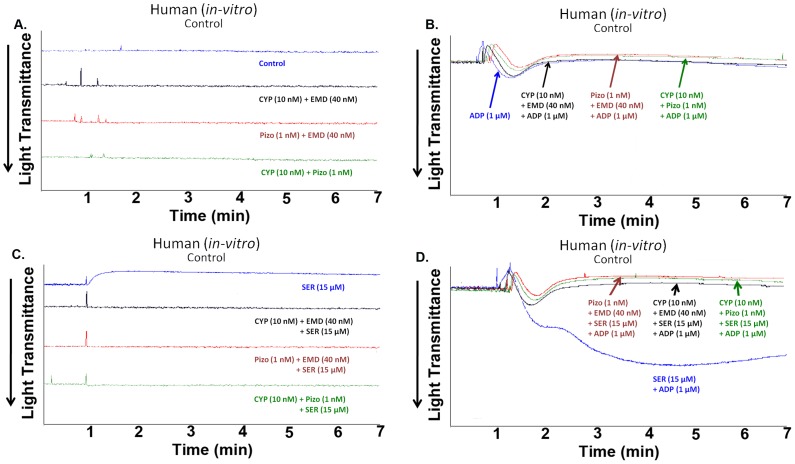
Combination of Cyproheptadine and pizotifen inhibits serotonin-enhanced ADP-induced human platelet aggregation *in vitro*. (A) Human PRP was treated with different combinations of cyproheptadine (10 nM), pizotifen (1 nM), and EMD 281014 (40 nM) in the absence of agonists. (B) Human PRP was pre-incubated with different combinations of cyproheptadine (10 nM), pizotifen (1 nM), and EMD 281014 (40 nM) for 1 min and activated with serotonin (15 µM). (C) Human PRP was pre-incubated with different combinations of cyproheptadine (10 nM), pizotifen (1 nM), and EMD 281014 (40 nM) for 1 min and activated with ADP (1 µM). (D) Human PRP was pre-incubated with different combinations of cyproheptadine (10 nM), pizotifen (1 nM), and EMD 281014 (40 nM) for 1 min and activated with ADP (1 µM) and serotonin (15 µM). Each experiment was repeated at least 3 times, with blood obtained from three separate donors.

In addition, combined drug effects of cyproheptadine (10 nM), pizotifen (1 nM), and EMD 281014 (40 nM) were examined in resting platelets and platelets stimulated with serotonin (15 µM) and/or ADP (1 µM). Our results indicated that none of the combinations of agents exerts any effect on resting platelets (**Fig. 3A**), or ADP-induced platelet aggregation (**Fig. 3B**). Expectedly though, these selective 5-HT_2A_ receptor antagonists did inhibit serotonin-induced platelet shape change (**Fig. 3C** **)**, and/or serotonin-enhanced platelet aggregation (**Fig. 3D**.

### Cyproheptadine and Pizotifen Inhibit Serotonin-enhanced ADP-induced Mouse Platelet Aggregation *in vitro*

**Figure 4:**
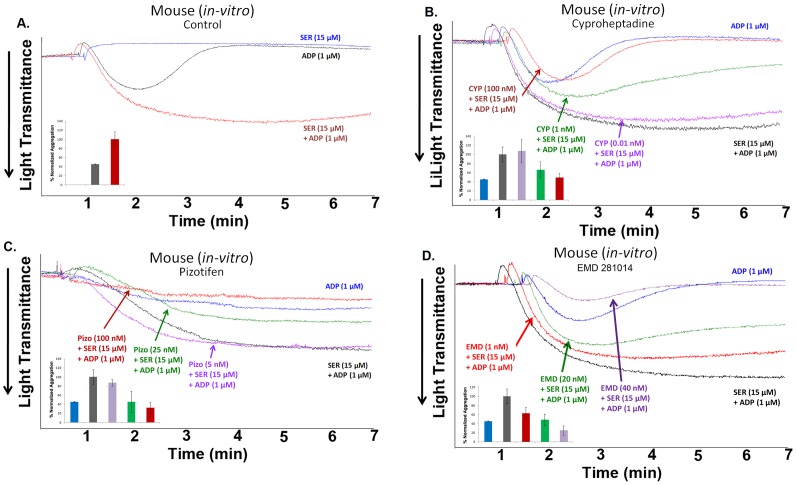
Cyproheptadine and pizotifen inhibit serotonin-enhanced ADP-induced mouse platelet aggregation *in vitro*. (A) Mouse PRP was stimulated with submaximal concentration of ADP (1 µM) in the presence or absence of serotonin (15 µM). (B) Mouse PRP was pre-incubated with increasing doses of cyproheptadine (0.01–100 nM) for 1 min and activated with ADP (1 µM) and serotonin (15 µM). (C) Mouse PRP was pre-incubated with increasing doses of pizotifen (5–100 nM) for 1 min and activated with 1 µM ADP and 15 µM serotonin. (D) Mouse PRP was pre-incubated with increasing doses of EMD 281014 (1–40 nM) for 1 min and activated with 1 µM ADP and 15 µM serotonin. Inset shows quantification of the data. Each experiment was repeated at least 3 times, with blood pooled from at least eight mice each time.

Similar platelet aggregation results were observed in mouse platelets *in vitro*. Simultaneous addition of serotonin (15 µM) with submaximal concentration of ADP (1 µM) significantly potentiated ADP-induced platelet aggregation in mouse platelets (**Fig. 4A**). Separate aggregation studies indicated that cyproheptadine (0.01–100 nM) and pizotifen (5–100 nM) dose-dependently inhibited serotonin-enhanced ADP-induced platelet aggregation (**Fig. 4B–4C**); similar findings were observed with EMD 281014 (1–40 nM; **Fig. 4D**). In summary, the 5-HT_2A_ receptor antagonists, cyproheptadine, pizotifen or EMD 281014, dose-dependently inhibited serotonin-enhanced platelet aggregation induced by submaximal concentration of ADP (1 µM) in both human and mouse platelets *in vitro*.

### Cyproheptadine and Pizotifen Inhibit Serotonin-enhanced ADP-induced Elevation in Intracellular Calcium in Human Platelets *in vitro*

**Figure 5:**
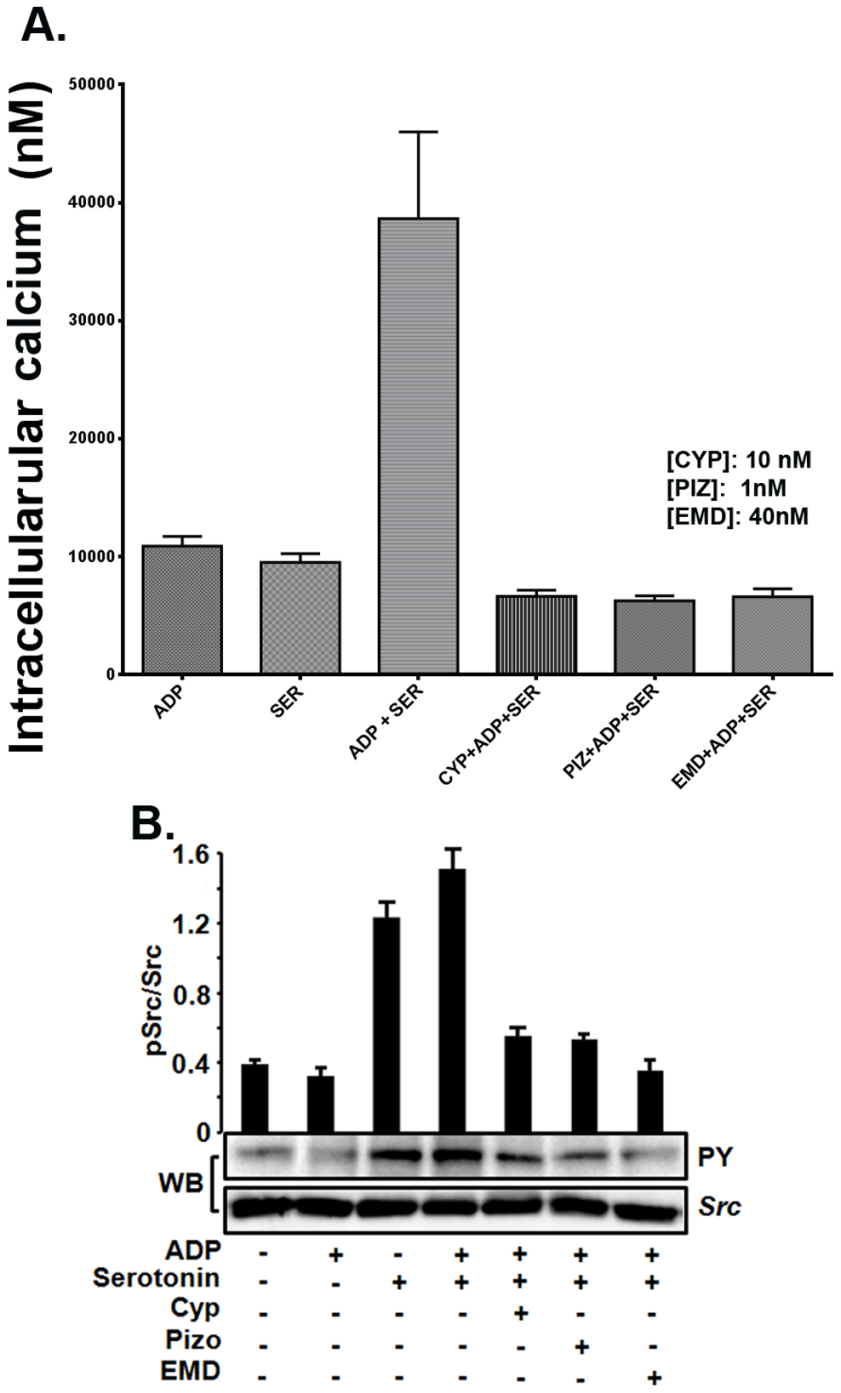
Cyproheptadine, and pizotifen inhibit intracellular calcium elevation and Src activation in human platelets *in vitro*. (A) Human platelets were loaded with Fura-2/AM to measure intracellular [Ca2+]_i_, in the presence or absence of Cyproheptadine (10 nM), pizotifen (1 nM) or EMD 281014 (40 nM) and activated with ADP (1 µM), serotonin (15 µM) or ADP and serotonin together. (B) Human platelets were incubated in the presence or absence of Cyproheptadine (10 nM), pizotifen (1 nM) or EMD 281014 (40 nM) for 5 minutes and then stimulated with ADP (1 µM), serotonin (15 µM) or ADP and serotonin together for 3 minutes, and subjected to immunoprecipitation followed by immunoblotting with anti-Src and anti-phosphotyrosine antibodies; upper panel shows quantification of the data using densitometry analysis. Each experiment was repeated at least 3 times, with blood obtained from three separate donors.

In additional control experiments, the capacity of these agents to inhibit the elevation in intercellular calcium that is serotonin-enhanced ADP-induced was also assessed. Our measurements revealed that cyproheptadine (10 nM), pizotifen (1 nM), and EMD 281014 (40 nM) all significantly reduced platelet intracellular calcium levels that are triggered by 1 µM ADP in the presence of 15 µM serotonin **(** **Fig. 5A**).

### Cyproheptadine and Pizotifen Inhibit Serotonin-enhanced ADP-induced Src Activation in Human Platelets *in vitro*

In another set of control experiments, western blot analysis was performed in order to assess the ability of cyproheptadine and pizotifen to inhibit serotonin-enhanced ADP-triggered tyrosine phosphorylation of Src family kinases. Analysis indicated that 5-HT_2A_ receptor antagonists, i.e., cyproheptadine (10 nM), pizotifen (1 nM), and EMD 281014 (40 nM) all have the ability to inhibit 15 µM serotonin-enhanced ADP-induced (1 µM) tyrosine phosphorylation in platelets, *in vitro* **(** **Fig. 5B**).

### Cyproheptadine and Pizotifen Inhibit Serotonin-enhanced ADP-induced Mouse Platelet Aggregation *ex vivo*

**Figure 6:**
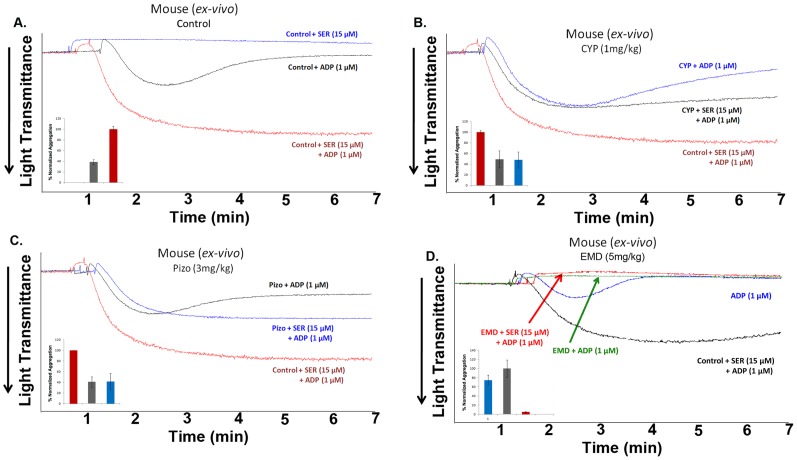
Cyproheptadine and pizotifen inhibit serotonin-enhanced ADP-induced mouse platelet aggregation *ex vivo*. (A) Mouse PRP obtained from vehicle-injected mice (once daily for 5 days) was stimulated with submaximal concentration of ADP (1 µM) in the presence or absence of serotonin (15 µM). (B) Mouse PRP obtained from cyproheptadine-injected mice (1 mg/kg IP once daily for 5 days) was stimulated with submaximal concentration of ADP in the presence or absence of serotonin. (C) Mouse PRP obtained from pizotifen-injected mice (3 mg/kg IP once daily for 5 days) was stimulated with submaximal concentration of ADP in the presence or absence of serotonin. (D) Mouse PRP obtained from EMD 281014 injected mice (5 mg/kg IP once daily for 5 days) was stimulated with submaximal concentration of ADP in the presence or absence of serotonin. Inset shows quantification of the data. Each experiment was repeated at least 3 times, with blood pooled from at least eight mice each time.

In order to investigate if the antiplatelet effects of cyproheptadine and pizotifen can be manifested under chronic dosing conditions in live animals, *ex vivo* mouse aggregation experiments were first performed. Using platelets isolated from mice injected with pharmacologically-relevant doses of 5-HT_2A_ receptor antagonists, once daily, for 5 days, our results demonstrated that, compared to the vehicle control (**Fig. 6A**), both cyproheptadine (1 mg/kg, IP) and pizotifen (3 mg/kg, IP) almost completely inhibited serotonin-enhanced ADP-induced platelet aggregation (**Fig. 6B, and 6C**). Similarly, chronic dosing with EMD 281014 (5 mg/kg, IP), inhibited serotonin-enhanced ADP-induced platelet aggregation (**Fig. 6D**), and (interestingly) exerted inhibitory effects on ADP-induced platelet aggregation, in the absence of serotonin (**Fig. 6D**). Together, our findings indicate that cyproheptadine and pizotifen’s antiplatelet effects are sustained following a chronic dosing regimen. It is noteworthy that the aforementioned doses and literature [29], [30], [32]–[38] guided our doses selection for the *in vivo* experiments, i.e., pharmacologically relevant doses.

### Cyproheptadine and Pizotifen Inhibit Serotonin-enhanced ADP-induced Phosphatidylserine Exposure, P-selectin Expression, and Glycoprotein IIb-IIIa Activation, in Human Platelets

**Figure 7:**
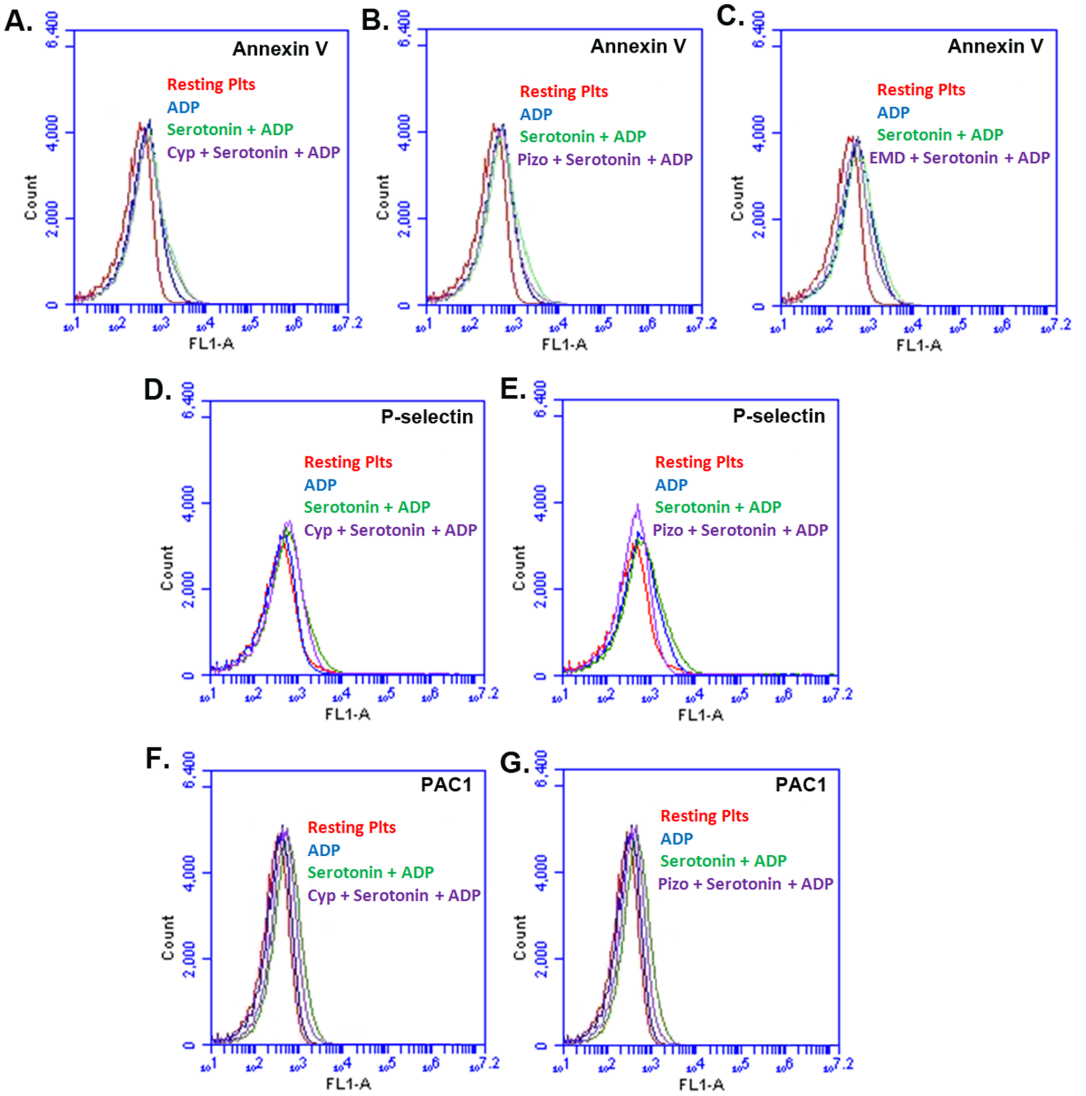
Cyproheptadine and pizotifen inhibit human platelet PS exposure (Annexin V), P-selectin, and GPIIb-IIIa (PAC-1 binding) activation *in vitro*. Washed platelets were incubated in the presence or absence of Cyproheptadine (10 nM), pizotifen (1 nM) or EMD 281014 (40 nM) for 5 minutes and then stimulated with ADP (1 µM), serotonin (15 µM) or ADP and serotonin together for 3 minutes. The reactions were stopped by fixing the platelets with 2% formaldehyde for 30 min at room temperature. (A–C): Platelets were incubated with FITC-conjugated Annexin V antibody, the fluorescent intensities were measured by flow cytometry, and the data were plotted as histogram. (D, E): Platelets were incubated with FITC-conjugated anti–P-selectin antibody, the fluorescent intensities were measured by flow cytometry, and the data were plotted as histogram. (F, G): Platelets were incubated with FITC-conjugated PAC-1 antibody, the fluorescent intensities were measured by flow cytometry, and the data were plotted as histogram. Each experiment was repeated at least 3 times, with blood obtained from three separate donors.

Next, we assessed whether the aforementioned 5-HT_2A_ receptor antagonists exert inhibitory effects on separate platelet functional responses, i.e., PS exposure, P-selectin expression, and GPIIb-IIIa activation, which are known to also be enhanced by serotonin. In platelets stimulated with submaximal concentration of ADP (1 µM) alone or ADP with serotonin (15 µM), an increase in mean fluorescence intensity (MFI), indicative of PS exposure, P-selectin expression, and GPIIb-IIIa activation, was observed (**Fig. 7A–G**). Moreover, in platelets pre-incubated with cyproheptadine (10 nM), and pizotifen (1 nM), resulted in a dramatic reversal of PS exposure, P-selectin expression, and GPIIb-IIIa activation that are ADP-stimulated serotonin potentiated, as follows: 1. Annexin V: 269.79±8.34 *versus* 226.94±8.05 for cyproheptadine; p<0.02; 275.64±8.42 versus 223.17±5.62 for pizotifen; p<0.01; 275.83±14.59 *versus* 210.41±76.73 for EMD 281014; p<0.02 **(** **Fig. 7A–C** **)**; 2. P-selectin: 933.35±81.61 *versus* 617.33±76.72 for cyproheptadine; p<0.02; 933.46±81.51 versus 624.40±95.84 for pizotifen **(** **Fig. 7D, 7E** **;** EMD 281014 data not shown); p<0.01; and 3. PAC1∶643.97±71.93 versus 576.77±58.39 for cyproheptadine; p<0.02; 643.97±71.93 versus 575.57±81.15 for pizotifen, p<0.02 (**Fig. 7F and 7G** **;** EMD 281014 data not shown). These data indicate that both antidepressant 5-HT_2A_ receptor antagonists have the capacity to inhibit serotonin-enhanced ADP-induced expression of multiple markers of platelet activation.

### Cyproheptadine and Pizotifen Prolong Occlusion Time in a Carotid Artery Injury-induced Thrombosis Model in Mice

**Figure 8:**
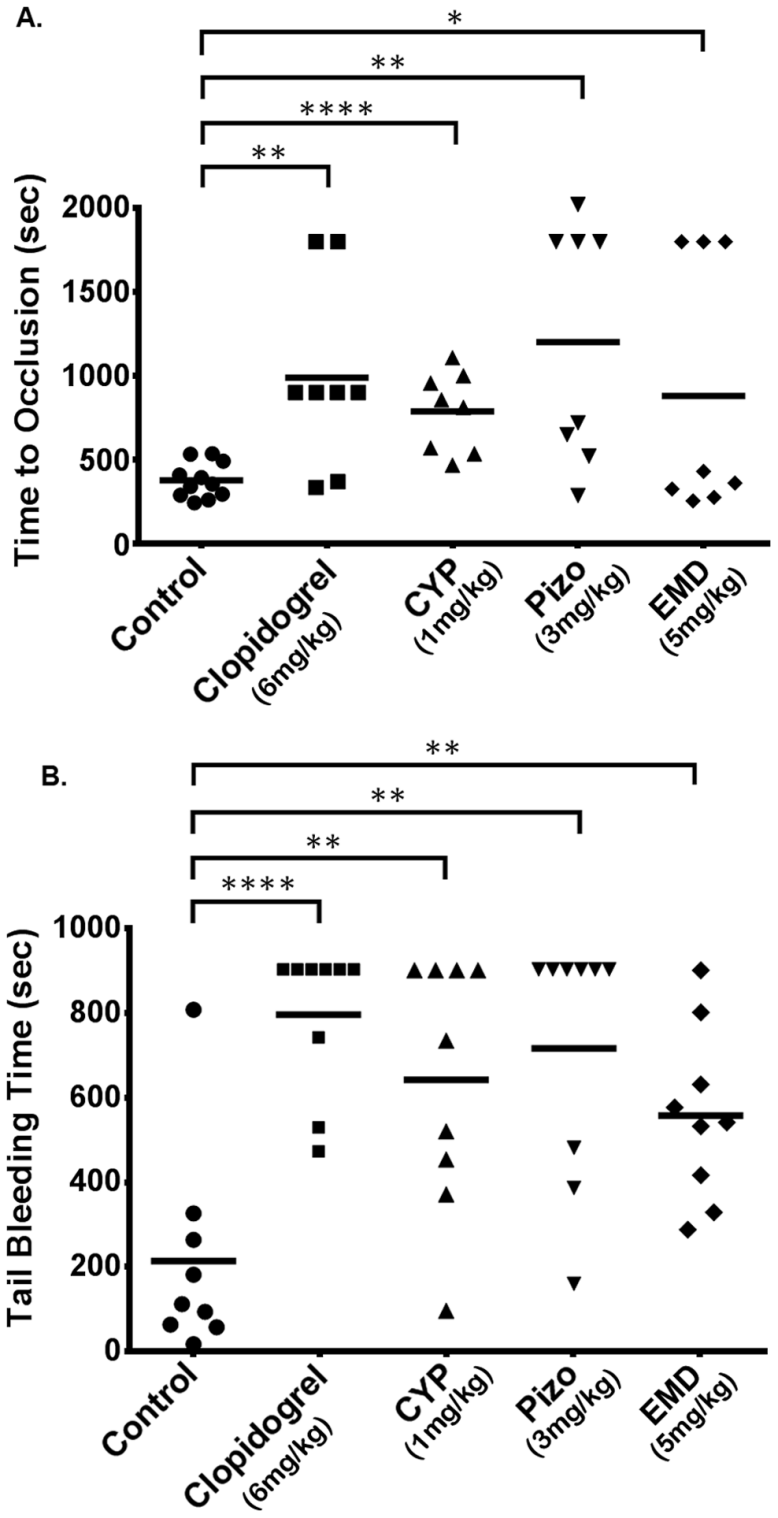
Cyproheptadine and pizotifen prolong occlusion times and tail bleeding times in mice. Each point represents the occlusion time or tail bleeding time of a single animal. Mice were treated IP with vehicle, 6/kg clopidogrel, 1 mg/kg cyproheptadine, 3 mg/kg pizotifen, or 5 mg/kg EMD 281014 once daily for 5 days before experiments. (A) Mean occlusion times for mice treated with: vehicle = 375.3±31.89 sec (n = 11), clopidogrel = 987.6±196.5 sec (n = 8), cyproheptadine = 787.4±84.08 sec (n = 8), pizotifen = 1199±253.1 sec (n = 8), and EMD 281014 = 879.9±270.0 sec (n = 8). **p<0.0022; ****p<0.0001; **p<0.0014; *p<0.0431. (B) Mean bleeding times for mice treated with: vehicle = 213.1±81.03 sec (n = 9), clopidogrel = 793.6±58.17 sec (n = 9), cyproheptadine = 643.1±97.78 sec (n = 9), pizotifen = 714.4±96.78 sec (n = 9), and EMD 281014 = 558.9±67.12 sec (n = 9). ****p<0.0001; **p<0.0038; **p<0.0011; **p<0.0047.

To evaluate the potential of cyproheptadine and pizotifen to alleviate thrombotic events *in vivo*, a mouse carotid artery FeCl_3_ injury model was used. Our studies revealed that mice treated with 1 mg/kg of cyproheptadine exhibited significantly increased time to occlusion as compared to vehicle-treated animals (787.4±84.08 sec *versus* 375.3±31.89 sec; mean, p<0.0001; **Fig. 8A**). Mice treated with 3 mg/kg of pizotifen also exhibited significant increase in time to vessel occlusion post-injury compared to control mice (1199±253.1 sec versus 375.3±31.89 sec; mean, p<0.0014). These data demonstrated that cyproheptadine and pizotifen are capable of delaying thrombus formation, and may be used to protect against arterial thrombosis.

In addition, mice treated with 5 mg/kg of EMD 281014 also exhibited significant increase time to occlusion as compared to vehicle-treated mice (879.9±270.0 sec versus 375.3±31.89 sec; mean, p<0.0431), which does not significantly differ from that for cyproheptadine- or pizptifen-treated mice. These data suggest that the antidepressant 5-HT_2A_ receptor antagonists, cyproheptadine and pizotifen, as well as EMD 281014 exert thromboprotective properties *in vivo*.

To provide a clinically relevant standard for comparing the effects of cyproheptadine and pizotifen on thromboprotection, the most widely prescribed antiplatelet agent clopidogrel was chosen. Our result revealed that time to vessel occlusion in mice treated with 6 mg/kg of clopidogrel is significantly prolonged compare to controls (987.6±196.5 sec versus 375.3±31.89 sec; mean, p<0.0022), but is comparable to those treated with 5-HT_2A_ receptor antagonists. This suggests that the antidepressant 5-HT_2A_ receptor antagonists are, perhaps, just as effective as the standard treatment of clopidogrel, at improving vessel occlusion time during a thrombotic event.

### Cyproheptadine and Pizotifen Prolong Tail Bleeding Time in Mice

We next examined the effect of cyproheptadine, pizotifen and EMD 281014 on hemostasis. Compared to vehicle-injected animals, mice injected with the 5-HT_2A_ receptor antagonists exhibited significant increase in tail bleeding times (vehicle = 213.1±81.03 sec; mean, CYP = 643.1±97.78 sec; mean, p<0.0038, Pizo = 714.4±96.78 sec; mean, p<0.0011; EMD 281014 = 558.9±67.12 sec; mean, p<0.0047; **Fig. 8B**). The prolongation of tail bleeding correlates to increased risk of impaired hemostasis, and this risk of bleeding is comparable to that associated with clopidogrel (793.6±58.17 sec; mean).

## Discussion

Cyproheptadine and pizotifen are two FDA-approved antidepressants that target and antagonize serotonin 5-HT_2A_ receptors in CNS, and have been commonly used to treat depressive disorders [27], [37]. In the periphery, 5-HT_2A_ receptors are located in vascular smooth muscles and platelets; and they have been implicated in regulatory processes including vasoconstriction and platelet aggregation [8], [15]. To this end, several potent and selective antagonists of the 5-HT_2A_ receptor were developed and tested and have demonstrated antiplatelet and antithrombotic activities [29], [39]–[44]. Unfortunately however, none of these molecules has made it into clinical practice, at least in the context of thrombogenesis [5], [9], [24]. Consequently, we decided to investigate whether the aforementioned antidepressant 5-HT_2A_ receptor antagonists may be repurposed for antiplatelet therapy [25], [26], [41].

Our initial *in vitro* aggregometry characterization revealed that cyproheptadine and pizotifen, have the capacity to dose-dependently inhibit serotonin-enhanced ADP-induced aggregation, but are less potent on mouse compared to human platelets. This is perhaps because the human and mouse 5-HT_2A_ receptor sequences, including their ligand binding domain(s), while highly conserved, are not identical, [45]. Nonetheless, the concentrations of either 5-HT_2A_ receptor antagonist needed to inhibit platelet aggregation in both human and murine platelets are in nM ranges, demonstrated a superior pharmacological profile.

The capacity of cyproheptadine, pizotifen and EMD 281014 to reverse the serotonin enhanced platelet function was further assessed using intracellular calcium measurements, and the activation of Src family kinases. It was found that these agents, at concentrations sufficient to almost completely inhibit serotonin-enhanced ADP-induced aggregation, also reduced intracellular calcium and Src activation. In separate control studies, the aforementioned 5-HT_2A_ receptor antagonists were also found to inhibit a host of platelet functional responses that are serotonin-enhanced ADP-induced, i.e., PS exposure, P-selectin expression, and GPIIb-IIIa activation. These findings indicated that cyproheptadine, pizotifen and EMD 281014 can down-regulate signal transduction associated with serotonin-enhanced platelet activation, and are consistent with the aggregation data. It appears that the magnitude of inhibition of GPIIb-IIIa activation by 5-HT_2A_ receptor antagonists does not correspond to their ability to inhibit aggregation, which we believe is due to experimental variability.

Given that patients are conventionally prescribed antidepressants or antiplatelet drugs for an extended period of time, we next sought to examine if the antiplatelet effects of these antidepressants can also be manifested in murine platelets under chronic dosing conditions. Thus, *ex vivo* experiments were performed following intraperitoneal (IP) injections of cyproheptadine (1 mg/kg) or pizotifen (3 mg/kg) once daily for 5 days. It is noteworthy that these doses were derived from the literature because of their pharmacological relevance for depressive disorders [29], [30], [32]–[36], to determine whether the same dose is capable of exerting dual pharmacological activity. Our data indicated that repeated injections with cyproheptadine and pizotifen did reverse serotonin-enhanced aggregation triggered by low-dose ADP.

To further determine if cyproheptadine and pizotifen’s antiplatelet properties can be translated to live animals, we performed mouse carotid artery thrombosis experiments. It was observed that the time to vessel occlusion drastically increased in mice treated with cyproheptadine or pizotifen. This finding indicates that these 5-HT_2A_ receptor antagonists have the ability to block serotonin-enhanced thrombogenesis, which derives from their ability to interfere with platelet activation. Clopidogrel was employed as a positive control, to provide a clinical benchmark for comparing the efficacy of cyproheptadine and pizotifen as alternative thromboprotective agents. Analysis revealed that while clopidogrel treatment did significantly prolonged occlusion times, its magnitude did not significantly differ from that observed in cyproheptadine-, and pizotifen- treated mice. Given their different target receptors (clopidogrel on P2Y_12_ receptors) these 5-HT_2A_ receptor antagonist may be able to serve as alternative medications, or complement to clopidogrel, given that dual therapy with clopidogrel is common clinical practice for thromboprotection [9], [46], [47].

In addition, to provide a relevant control for antidepressant 5-HT_2A_ receptor antagonists cyproheptadine and pizotifen, EMD 281014 was chosen for comparison. Analysis revealed that while EMD 281014 also has the capacity to prolong occlusion times, large biological variations were observed. Given that EMD 281014, cyproheptadine and pizotifen are all 5-HT_2A_ receptor antagonists, but lower doses of cyproheptadie and pizotifen were needed to achieve higher significant prolongation of occlusion times and more consistently with less variation, this suggests, cyproheptadine and pizotifen may be more predictable and effective antiplatelet agents compared to EMD 281014.

Increased risk of bleeding is a common concern for patients on any antiplatelet agent, and thus it was investigated using cyproheptadine and pizotifen. Our results indicated that the tail bleeding times were significantly increased in animals treated with either cyproheptadine or pizotifen; similar to what was observed in mice treated with clopidogrel and EMD 281014. These data indicate that continuous treatment with cyproheptadine or pizotifen may result in increased bleeding risk, similar to the (currently) most commonly-prescribed antiplatelet drug clopidogrel. Given that cyproheptadine and pizotifen’s antithrombotic effects are comparable to clopidogrel, we believe that depressive patients with primed platelet activity or high risk of thrombosis will likely benefit from one of these agents. Repurposing old drugs for new applications can be advantageous as this approach could dramatically reduce the cost and time required for drugs to be approved for market and used in clinics. Furthermore, if modeled over their chemistry, structural analogs or derivatives of these 5-HT_2A_ receptor antagonists may be developed, with much more competitive pharmacological profiles. Lastly, while our data may argue against it, we cannot exclude contribution from vasculature or other 5-HT_2A_ receptors to the observed antiplatelet phenotype.

In summary, our studies demonstrate that FDA-approved antidepressants, 5-HT_2A_ receptor antagonists pizotifen and cyproheptadine (at standard dosages), exert antiplatelet activity, and thus can be repurposed for the treatment of thromboembolic disorders; albeit their use may interfere with normal hemostasis.

## Materials and Methods

The human donor blood part of the work has been approved by the Institutional Review Board at Western University of Health Sciences, and donors were asked to sign a written consent, and a subjects’ bill of rights. The animal work part of these studies has been approved by Institutional Animal Care and Use Committee at Western University of Health Sciences.

### Reagents and Materials

Serotonin hydrochloride, pizotifen and ADP were obtained from Sigma Aldrich (St. Louis, MO), cyproheptadine and EMD 281014 were obtained from Tocris Bioscience (Bristol, UK), clopidogrel was purchased from LKT Laboratories, Inc. (St. Paul, MN), stir bars and other disposables were from Chrono-Log (Havertown, PA), and U46619 was obtained from Cayman Chemical (Ann Arbor, MI). Src antibody, FITC-conjugated Annexin V, anti–P-selectin, and PAC-1 antibodies were purchased from Cell Signaling Technology, Inc. (Danvers, MA). The anti-phosphotyrosine antibody was from BD Biosciences, (Franklin Lakes, NJ). Fura-2 acetoxymethyl ester (fura-2/AM) and Pluronic® F-127 were from Invitrogen (Grand Island, NY). The C57BL/6 mice were obtained from Jackson laboratory (Bar Harbor, ME). Platelet count was determined using an automated hematology analyzer (Drew Scientific Dallas, TX).

### Animals

C57BL/6 mice were obtained from Jackson Laboratory (Bar Harbor, ME). Mice were housed in groups of 1–4 at 24°C, under 12/12 light/dark cycles, with access to water and food ad libitum. All experiments involving animals were perform in compliance with the institutional guidelines, and were approved by the Western University of Health Sciences Institutional Animal Care and Use Committee.

### Human and Murine Platelet Preparation

Blood was drawn from healthy volunteers who denied taking any medication for 1 week prior to collection, or from C57BL/6 mice (8–10 weeks old). Mice were anesthetized and blood was collected from the heart. Coagulation was inhibited by 3.8% w/v sodium citrate solution (1 part sodium citrate to 9 parts blood). Human or mouse platelet rich plasma (PRP) was obtained by centrifugation at room temperature. Platelets were counted with automated hematology analyzer and their count adjusted to 7×10^7^ platelets/ml, prior to each experiment.

Washed human platelets were prepared as described in Karim et al [48]. PRP was isolated in the presence of 0.37 units/ml apyrase and 10 ng/ml PGI_2_ by centrifugation at 150×*g* for 10 min at 20°C. PRP was centrifuged at 900×*g* for 10 min, and pelleted platelets were resuspended in HEPES/Tyrode’s buffer (20 mMHEPES/NaOH, pH 6.5, 128 mM NaCl, 2.8 mM KCl, 1 mM MgCl_2_, 5 mM D-glucose, 12 mM NaHCO_3_, 0.4 mM NaH_2_PO_4_) containing 1 mM EGTA, 0.37 units/ml apyrase, and 10 ng/ml PGI_2_. Platelets were washed and resuspended in HEPES/Tyrode’s buffer (pH 7.4) without EGTA, apyrase, or PGI_2_. The final platelet counts were adjusted to 4×10^8^ platelets/ml, unless otherwise indicated.) PRP was isolated in the presence of apyrase (0.37 U/mL) and PGI_2_ (10 ng/mL) by centrifugation at 150×*g* for 10 minutes at RT. PRP was centrifuged at 900×*g* for 10 minutes and platelets were resuspended in HT containing 1 mM EGTA, apyrase, and PGI_2_. Platelets were washed and resuspended in HT (pH 7.4) without EGTA, apyrase, or PGI_2_.

### *In vitro* Platelet Aggregation

PRP was incubated with 5HT_2A_ receptor antagonists, cyproheptadine, pizotifen, or EMD 281014 for 1 min prior to experiments, except in control experiments. Platelets were activated with submaximal concentration of ADP (1 µM), in the presence or absence of 15 µM serotonin. Platelet aggregation was measured by the turbidometric method using model 490 aggregometer (Chrono-Log Corporation, Havertown, PA). Each experiment was repeated at least 3 times, with blood collected from three different human donors, or pooled together from at least eight mice for each experiment. (what about in vitro?).

### *Ex vivo* Platelet Aggregation

Mice were injected with vehicle (DMSO), or pharmacologically/therapeutically relevant doses [29], [30], [32]–[38] of cyproheptadine (1 mg/kg), pizotifen (3 mg/kg), clopidogrel (6 mg/kg), or EMD 281014 (5 mg/kg) using the intraperitoneal (IP) route once daily for 5 days; in an attempt to mimic chronic administration of these drugs in patients. Mice were sacrificed two hours post last injection, and their blood collected. Platelets (with counts adjusted as described before) were stimulated with 1 µM ADP in the presence or absence of 15 µM serotonin, and platelet aggregation was measured. Each experiment was repeated at least 3 times, with blood pooled from at least eight mice each time.

### Intracellular Calcium Measurement in Platelets

Intra-platelet calcium was measured using Fura-2-acetoxymethyl ester (Fura-2AM) as described [48]. Mouse platelets (2.0×10^8^/mL) were labeled with 12.5 µM Fura-2AM and 0.2% Pluronic F-127 in HEPES/Tyrode buffer (pH 7.4) for 45 min at 37°C. After washing, the platelets were resuspended without apyrase to a concentration of 2.0×10^8^/mL. Samples (1 mL) were added to siliconized cuvettes, recalcified with 0.7 mM CaCl_2_, and incubated in the presence or absence of cyproheptadine (10 nM), pizotifen (1 nM) or EMD 281014 (40 nM) for 5 minutes and then stimulated with ADP (1 µM), serotonin (15 µM) or ADP and serotonin together for 3 minutes with constant stirring. Fluorescence was analyzed by excitation at 340 nm and 380 nm and emission was measured at 509 nm using a model LS50B Luminescence Spectrometer (Perkin-Elmer Instruments, Shelton, CT). The ratio of fura-2 emissions was calculated simultaneously using FL WinLab software and converted to [Ca^2+^]_i_, as described previously [49].

### Immunoprecipitation and Immunoblotting

Immunoprecipitation was carried out as described in Karim et al [48]. Briefly, human platelets were incubated in the presence or absence of Cyproheptadine (10 nM), pizotifen (1 nM) or EMD 281014 (40 nM) for 5 minutes and then stimulated with ADP (1 µM), serotonin (15 µM) or ADP and serotonin together for 3 minutes followed by lysis with 2× lysis buffer (40 mM Tris-HCl [pH 7.5], 150 mM NaCl, 2 mM Na_2_EDTA, 2 mM EGTA, 2% Triton X-100, 2% sodium deoxycholate, 5 mM sodium pyrophosphate, 2 mM Na_3_VO_4_, and protease inhibitor cocktail). Platelet lysates were clarified by centrifugation and the supernatants were precleared by incubating with rabbit IgG and then incubated with anti-Src antibody. Immunoprecipitates were separated by sodium dodecyl sulfate-polyacrylamide gel electrophoresis (SDS-PAGE) and transferred to Immobilon-P PVDF membranes (Bio-Rad, Hercules, CA). They were then probed with the primary antibodies (Src and anti-phosphotyrosine) and visualized with horseradish peroxidase-labeled anti-rabbit IgG or anti-mouse IgG as required. The antibody binding was detected using enhanced chemiluminescence substrate (Thermo Scientific, Rockford, IL). Images were obtained with ChemiDoc MP Imaging System (Bio-Rad, Hercules, CA) and quantified with Image Lab software Version 4.1 (Bio-Rad, Hercules, CA).

### Flow Cytometric Analysis

Human platelets (2×10^8^) were incubated in the presence or absence of Cyproheptadine (10 nM), pizotifen (1 nM) or EMD 281014 (40 nM) for 5 minutes and then stimulated with ADP (1 µM), serotonin (15 µM) or ADP and serotonin together for 3 minutes. The reactions were stopped by fixing the platelets with 2% formaldehyde for 30 min at room temperature. Finally, platelets were incubated with FITC-conjugated Annexin V, anti–P-selectin, or PAC-1 antibodies at room temperature for 30 min in the dark. Finally, the platelets were diluted 2.5-fold with HEPES/Tyrode buffer (pH 7.4). The samples were transferred to FACS-tubes and fluorescent intensities were measured using a BD Accuri C6 flow cytometer and analyzed using CFlow Plus (BD Biosciences, Franklin Lakes, NJ).

### *In vivo* Thrombosis Model

These studies were performed as described previously [50]. Briefly, mice 8–10 weeks old received IP injections of cyproheptadine, pizotifen, clopidogrel, or EMD 281014 at doses described before, and were anesthetized with isoflurane. Then, the left carotid artery was exposed and cleaned, and baseline carotid artery blood flow was measured with Transonic micro-flowprobe (0.5 mm, Transonic Systems Inc., Ithaca, NY). After stabilization of blood flow, 7.5% ferric chloride (FeCl_3_) was applied to a filter paper disc (1-mm diameter) that was immediately placed on top of the artery for 3 min. Blood flow was continuously monitored for 30 min, or until blood flow reached stable occlusion (zero blood flow for 2 min). Data was recorded and time to vessel occlusion was calculated as the difference in time between stable occlusion and removal of the filter paper (with FeCl_3_). An occlusion time of 30 min was considered as the cut-off time for the purpose of statistical analysis.

### Tail Bleeding Time

Mice were IP injected with cyproheptadine, pizotifen, clopidogrel, EMD 281014 or vehicle once daily for 5 days, as described before. Hemostasis was examined using the tail transection technique [50]. Briefly, mice were anesthetized and placed on a 37°C homeothermic blanket. Tail was transected 5 mm from the tip using a sterile scalpel. After transection, the tail was immediately immersed in saline (37°C, constant temperature) and the time to bleeding cessation was measured. Bleeding time of 15 min was considered as the cut-off time for the purpose of statistical analysis.

### Statistical Analysis

All experiments were performed at least three times. Analysis of the data was performed using GraphPad PRISM statistical software (San Diego, CA) and presented as mean ± SEM. The Mann-Whitney test was used for the evaluation of differences in mean occlusion and bleeding times. Analysis was also conducted using t-test, and similar results were obtained. Significance was accepted at P<0.05 (two-tailed P value), unless stated otherwise.
